# Association Between MIND Diet Adherence and Mortality: Insights from Diabetic and Non-Diabetic Cohorts

**DOI:** 10.1038/s41387-023-00247-1

**Published:** 2023-10-10

**Authors:** Yanjun Song, Zhen’ge Chang, Chenxi Song, Kongyong Cui, Boqun Shi, Rui Zhang, Qiuting Dong, Kefei Dou

**Affiliations:** 1 Cardiometabolic Medicine Center, Fuwai Hospital, National Center for Cardiovascular Diseases, Chinese Academy of Medical Sciences and Peking Union Medical College, 167A Beilishi Road, Xi Cheng District, Beijing, 100037 China; 2 State Key Laboratory of Cardiovascular Disease, Beijing, China; 3 Department of Respiratory Medicine, Civil Aviation General Hospital, Beijing, China

**Keywords:** Type 2 diabetes, Nutrition

## Abstract

**Background/Objectives:**

To date, evidence regarding the protective roles of the Mediterranean-Dietary Approaches to Stop Hypertension Intervention for Neurodegenerative Delay (MIND) diet in patients with type 2 diabetes mellitus (T2DM) is scarce. This study aims to estimate the impact of adhering to the MIND diet on the mortality in patients with and without T2DM.

**Subjects/Methods:**

In this cohort study, 6887 participants (1021 patients with T2DM) from the NHANES dataset were analyzed. The exposure is the MIND diet adherence. The primary outcomes are all-cause and cardiovascular (CV) deaths.

**Results:**

We documented 1087 all-cause deaths consisting of 377 CV deaths during the follow-up (median time of 10 years). Among participants with T2DM, those with a high MIND score (> 8.0, range of MIND score: 4.5–13) had a significantly lower risk of all-cause death (hazard ratio [HR] 0.75, 95% confidence interval [CI]: 0.59, 0.96, *P* = 0.021) and CV death (HR 0.50, 95% CI: 0.29, 0.87, *P* = 0.014) compared to those with a low MIND score (≤ 8.0). In participants without T2DM, a high MIND score was associated with a significant decrease in the risk of all-cause death (HR = 0.83, 95% CI: 0.70, 0.99, *P* < 0.001), but the association with CV death risk was not statistically significant.

**Conclusion:**

This study uncovered significant associations between the MIND diet and decreased risk of all-cause and CV death in patients with T2DM. The findings highlight the potential benefits of following the MIND diet in managing and enhancing the outcomes of individuals with T2DM.

## Introduction

Type 2 diabetes mellitus (T2DM) is a well-recognized public health concern associated with significant morbidity and mortality rates [1]. Given the projected increase in the global prevalence of DM to approximately 552 million cases by 2030, there is a pressing need to improve the prognosis for individuals affected by this condition [2]. Whole grains, fruits, and vegetables can reduce the risk of developing diabetes and its related complications by improving insulin and glucose metabolism [3, 4]. Furthermore, certain dietary patterns, such as the Mediterranean (MED) diet and a vegetarian diet, have been shown to be beneficial in the prevention and management of DM, as they enhance insulin sensitivity and glycemic control [5, 6]. Therefore, there is a growing interest in further investigating the impact of healthy eating habits with a strong emphasis on diabetes prevention.

The Mediterranean-Dietary Approach to Stop Hypertension Intervention for Neurodegenerative Delay (MIND) diet is an innovative dietary approach that combines elements from the MED diet and the DASH diet [7–10]. The MIND diet incorporates these principles and adds a specific focus on limiting foods that contribute to the pathogenesis and development of DM, such as fried/fast foods, sweets, butter, and margarine. The MIND diet has demonstrated significant protective effects in enhancing cognitive performance and preventing dementia [11, 12]. By combining the beneficial components of the MED and DASH diets with a targeted approach to prevent DM, the MIND diet offers a comprehensive dietary strategy for promoting cognitive well-being. In addition, the MIND diet also emphasizes green leafy vegetables and berries, which not only confer brain-protective effects but also attenuate cardiovascular (CV) diseases [13, 14] and glycemic metabolism disorders [15, 16]. Therefore, exploring the metabolic benefit of the MIND diet in patients with DM is a topic of significant interest and importance. However, evidence regarding the impact of the MIND diet on the prognosis of patients with T2DM is still scarce.

In this study, we enrolled 6887 participants in the U.S. National Health and Nutrition Examination Survey (NHANES), 1021 of whom had T2DM. We aim to evaluate the impact of the MIND diet on life expectancy among patients with and without T2DM.

## Materials/Subjects and Methods

### Study population

**Fig. 1:**
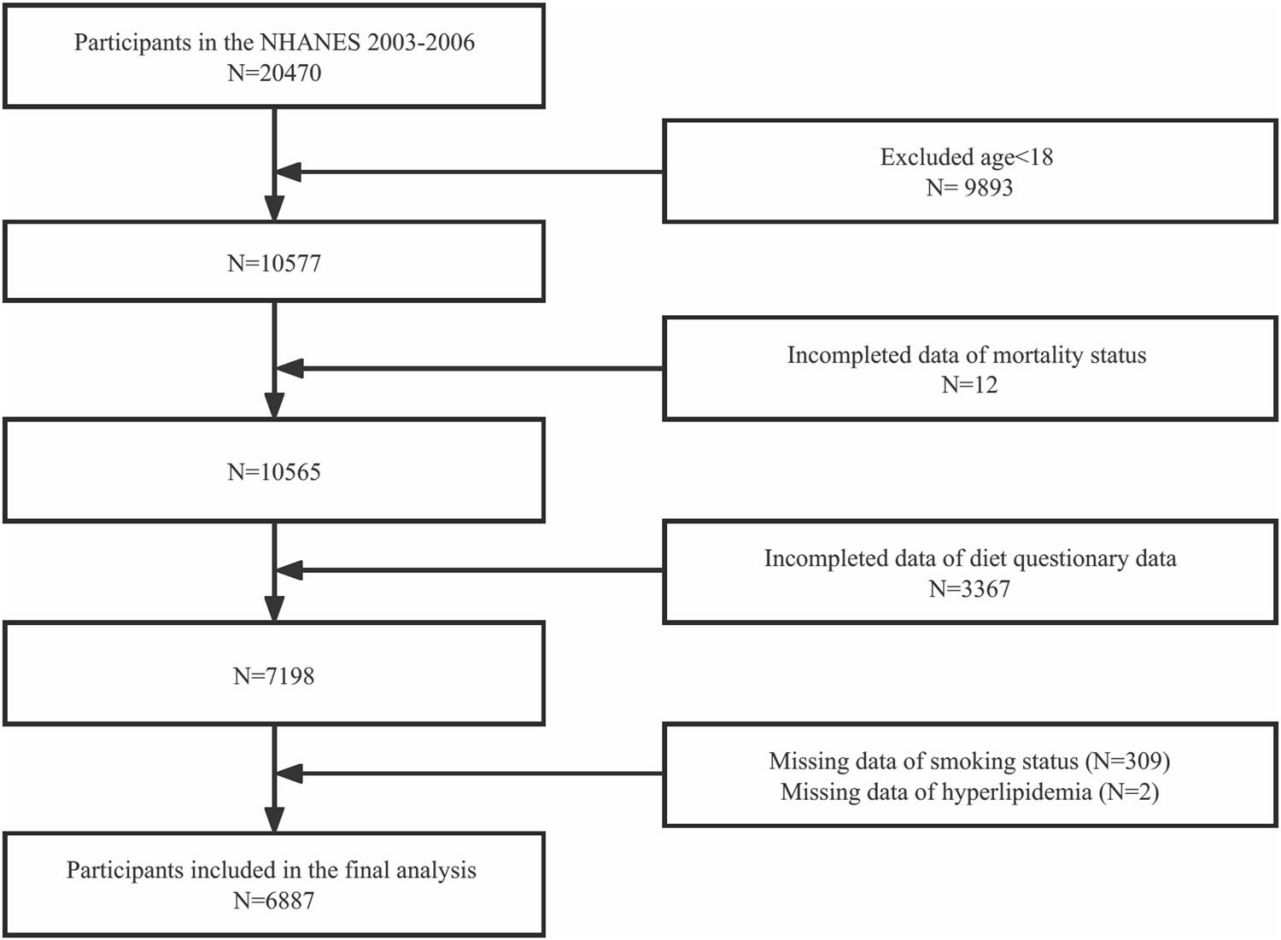
Flowchart of participant selection. NHANES National Health and Nutrition Examination Survey.

The data from NHANES 2003 to 2006 was utilized in this study. NHANES 2003–2006 initially enrolled 20470 individuals in total. We included 6887 people in the final analysis after eliminating participants under the age of 18 (*n* = 9893), without mortality data (*n* = 12), without diet data (*n* = 3367), without smoking status (*n* = 309), and with a diagnosis of hyperlipidemia (*n* = 2). A complete flowchart of the procedure for choosing study participants is shown in Fig. 1. The participants were separated into four groups based on whether or not they had type 2 diabetes (low MIND score/non-DM, high MIND score/non-DM, low MIND score/DM, and high MIND score/DM) after being divided into two groups based on their MIND scores (low score [≤ 8.0] and high score [> 8.0]). The median of all participants (MIND score = 8.0) was established as the ideal MIND score cutoff. T2DM was diagnosed with the standard criterion [17].

### MIND diet scoring

The MIND score was used to assess how well the MIND diet was followed. The MIND diet consisted of five harmful food groups and ten beneficial food groups. The scores of all 15 components were added together to create the final MIND score [15]. During the 2003–2006 cycles, we gathered information on each food element pertinent using the Food Frequency Questionnaire (FFQ). Text S1 provides further details on the FFQ. Table S1 shows how the MIND scores were specifically determined. These participants’ MIND scores varied from 4.5 to 13 points (the typical range is 0 to 15 points).

### Follow-up and outcomes

Research follow-up was provided to participants for a median of 10 years. The all-cause and CV deaths were the study’s main outcomes. Deaths from CV illnesses or cerebrovascular diseases were referred to as “CV death”.

### Statistical analysis

Following the analytical guidelines provided by NHANES, our study accounted for sample weights, clustering, and stratification to ensure the generalizability of our findings to the entire U.S. population aged 18 years and above. Using the ANOVA and the χ2 test for continuous and categorical variables respectively, we examined the differences between the groups. To include all data for modeling, imputation was employed with the median for the variables with missing rates lower than 5%. Values with a missing rate higher than 5% were assigned to a separate “Unknown” category. To estimate the HRs and 95% CIs, we utilized muti-variable Cox models. Three models were included in the analysis, progressively adjusting for potential covariates. Age, sex, and race/ethnicity modifications were all incorporated in Model 1. Model 2 included further adjustments for education level, smoking status, the percentage of families with incomes below the poverty line, body mass index (BMI), and physical activity. Model 3, the fully adjusted model, incorporated additional adjustments for hypertension, hyperlipidemia, energy intake, and estimated glomerular filtration rate (eGFR). Concrete details regarding each covariate can be found in Text S2. Kaplan-Meier (K-M) plots were used for the survival analysis, and the Log-rank test was used to determine the data’s statistical significance.

We further performed subgroup analyses by demographic characteristics (including age, sex, and race), lifestyles (including smoking status and physical activity), BMI, and renal function [eGFR]. Each sensitivity analysis separately excluded participants who were non-Hispanic Black, died within a year of the follow-up, whose family income to poverty ratio was unknown, and participants with heart failure, ischemic heart disease (IHD, including myocardial infarction [MI], and angina), cerebral disease, dual antiplatelet therapy, statin therapy, and hypoglycemic treatment.

We conducted all statistical analyses using R version 4.1.3. A *p*-value less than 0.05 (two-tailed) was considered statistically significant in our study. The code used during the current study is available from the corresponding author on reasonable request.

## Results

### Characteristics of the study population

This research comprised 6887 individuals in all. By high or low MIND scores, Table 1 groups the general population’s baseline characteristics. Participants in the high MIND score group were older, tend to be white people, non-Hispanic women, and smokers than those in the low MIND score group. Also, those with high MIND scores had lower levels of calorie consumption, waist circumference, and physical activity, as well as greater levels of education and family income.

**Table 1:** Baseline characteristics of participants according to their MIND diet score.

		MIND diet score		
Characteristics	Total (*N* = 6887)	Low (*N* = 3846)	High (*N* = 3041)	*P*-value
Age (years)	47.13 ± 0.45	45.04 ± 0.43	49.66 ± 0.61	< 0.0001
Sex, *n* (%)				< 0.0001
Male	3181(46.19)	1927(50.80)	1254(39.13)	
Female	3706(53.81)	1919(49.20)	1787(60.87)	
Race/ethnicity, *n* (%)				< 0.001
Non-Hispanic White	3809(55.31)	2073(70.52)	1736(75.53)	
Non-Hispanic Black	1333(19.36)	847(13.64)	486(8.67)	
Mexican American	1272(18.47)	671(7.74)	601(8.20)	
Others	473(6.87)	255(8.10)	218(7.60)	
Education level, *n* (%)				< 0.0001
Less than high school	1831(26.59)	1143(19.48)	688(12.50)	
High school or equivalent	1730(25.12)	1079(30.83)	651(21.49)	
College or above	3326(48.29)	1624(49.68)	1702(66.01)	
Family income to poverty ratio, *n* (%)				< 0.0001
< 1	1077(15.64)	679(13.06)	398(8.82)	
≥ 1 & < 3	2781(40.38)	1636(37.41)	1145(31.55)	
≥ 3	2715(39.42)	1352(45.02)	1363(56.08)	
Unknown	314(4.56)	179(4.51)	135(3.55)	
Smoking status, *n* (%)				< 0.0001
Never	3526(51.2)	1887(46.69)	1639(53.92)	
Former	1920(27.88)	963(22.55)	957(30.43)	
Current	1441(20.92)	996(30.76)	445(15.65)	
BMI (kg/m^2^), *n* (%)				0.07
< 25.0	2082(30.71)	1141(33.70)	941(33.80)	
25.0–29.9	2356(34.75)	1274(31.64)	1082(34.85)	
≥ 30.0	2341(34.53)	1370(34.66)	971(31.35)	
Physical activity, *n* (%)				< 0.0001
Sedentary	1774(25.76)	1114(21.93)	660(15.03)	
Insufficient	2621(38.06)	1454(41.57)	1167(41.41)	
Moderate	1129(16.39)	591(16.74)	538(19.04)	
High	1363(19.79)	687(19.77)	676(24.52)	
Diabetes, *n* (%)	1021(14.83)	581(11.04)	440(10.78)	0.79
Hypertension, *n* (%)	2984(43.33)	1687(38.06)	1297(38.27)	0.92
Hyperlipidemia, *n* (%)	4954(71.93)	2775(70.09)	2179(69.96)	0.93
Heart failure	264(3.83)	166(2.87)	98(2.34)	0.37
Ischemic heart disease	498(7.24)	289(5.81)	209(5.10)	0.32
Myocardial infarction	361(5.24)	211(4.25)	150(3.65)	0.63
TC (mg/dL)	200.23 ± 0.70	198.60 ± 0.76	202.20 ± 1.25	0.02
TG (mg/dL)	142.74 ± 2.53	145.97 ± 3.38	138.86 ± 4.20	0.22
LDL-C (mg/dL)	115.77 ± 0.84	115.81 ± 1.25	115.72 ± 1.33	0.96
HbA1C (%)	5.47 ± 0.02	5.47 ± 0.02	5.46 ± 0.02	0.48
Fasting glucose (mg/dL)	5.65 ± 0.04	5.63 ± 0.05	5.68 ± 0.06	0.38
eGFR (mL/min/1.73 m^2^)	93.13 ± 0.66	94.95 ± 0.60	90.91 ± 0.84	< 0.0001
Waist circumference (cm)	97.53 ± 0.45	98.21 ± 0.47	96.71 ± 0.63	0.03
Energy intake (Kcal)	2117.02 ± 15.62	2159.73 ± 17.75	2065.08 ± 23.42	0.001
DAPT	8(0.12)	4(0.09)	4(0.04)	0.40
Statin therapy	1073(15.59)	576(13.13)	497(13.95)	0.45
Hypoglycemic treatment	630(9.16)	357(6.62)	273(6.58)	0.94

Data are presented as weighted means ± SEs for continuous variables and unweighted numbers (weighted percentages) for categorical variables.

*MIND diet* Mediterranean-DASH Diet Intervention for Neurodegenerative Delay diet, *BMI* Body mass index, *TC* Total cholesterol, *TG* Triglyceride, *LDL-C* Low-density lipoprotein cholesterol, *HDL-C* High-density lipoprotein cholesterol, *HbA1c* Glycosylated hemoglobin, type A1C; *eGFR* estimated glomerular filtration rate, *DAPT* Dual antiplatelet therapy.

The baseline characteristics of four groups (low MIND score/non-DM, high MIND score/non-DM, low MIND score/DM, and high MIND score/DM) are presented in Table 2. Compared with people in the low MIND score/non-DM group, those in the other three groups were older, more likely to be female and smokers, and had a higher prevalence of hypertension, hyperlipidemia, heart failure, IHD, MI, and medical therapies of hypoglycemic treatment, DAPT, and statin treatment.

**Table 2:** Baseline characteristics of participants according to their MIND diet score and DM status.

Characteristics	Total (*N* = 6887)	MIND diet score -H/non-DM (*N* = 2601)	MIND diet score -L/non-DM (*N* = 3265)	MIND diet score -H/DM (*N* = 440)	MIND diet score -L/DM (*N* = 581)	*P*-value
Age (years)	47.13 ± 0.45	48.25 ± 0.59	43.31 ± 0.42	61.29 ± 0.83	59.00 ± 1.05	< 0.0001
Sex, *n* (%)						< 0.0001
Male	3181(46.19)	1046(38.25)	1626(50.80)	208(46.37)	301(50.80)	
Female	3706(53.81)	1555(61.75)	1639(49.20)	232(53.63)	280(49.20)	
Race/ethnicity, *n* (%)						< 0.0001
Non-Hispanic White	3809(55.31)	1515(75.62)	1831(71.90)	221(74.80)	242(59.40)	
Non-Hispanic Black	1333(19.36)	410(8.36)	672(12.74)	76(11.24)	175(20.92)	
Mexican American	1272(18.47)	482(8.16)	542(7.64)	119(8.48)	129(8.53)	
Others	473(6.87)	194(7.86)	220(7.72)	24(5.47)	35(11.15)	
Education level, *n* (%)						< 0.0001
Less than high school	1831(26.59)	537(11.47)	911(18.25)	151(20.97)	232(29.41)	
High school or equivalent	1730(25.12)	539(20.66)	920(30.85)	112(28.37)	159(30.71)	
College or above	3326(48.29)	1525(67.86)	1434(50.90)	177(50.66)	190(39.88)	
Family income to poverty ratio, *n* (%)						< 0.0001
< 1	1077(15.64)	330(8.67)	556(12.80)	68(10.12)	123(15.17)	
≥ 1 & < 3	2781(40.38)	956(30.71)	1357(36.30)	189(38.48)	279(46.35)	
≥ 3	2715(39.42)	1204(57.09)	1203(46.57)	159(47.75)	149(32.52)	
Unknown	314(4.56)	111(3.53)	149(4.33)	24(3.65)	30(5.95)	
Smoking status, *n* (%)						< 0.0001
Never	3526(51.2)	1426(54.16)	1616(46.92)	213(51.98)	271(44.81)	
Former	1920(27.88)	782(29.56)	764(21.48)	175(37.60)	199(31.21)	
Current	1441(20.92)	393(16.28)	885(31.60)	52(10.42)	111(23.97)	
BMI (kg/m^2^), *n* (%)						< 0.0001
< 25.0	2082(30.71)	883(36.60)	1055(36.15)	58(10.38)	86(13.67)	
25.0–29.9	2356(34.75)	927(35.25)	1114(32.14)	155(31.49)	160(27.51)	
≥ 30.0	2341(34.53)	757(28.15)	1053(31.70)	214(58.13)	317(58.81)	
Physical activity, *n* (%)						< 0.0001
Sedentary	1774(25.76)	518(13.65)	877(20.22)	142(26.50)	237(35.64)	
Insufficient	2621(38.06)	1011(41.59)	1275(42.24)	156(39.90)	179(36.19)	
Moderate	1129(16.39)	469(19.62)	515(17.05)	69(14.22)	76(14.22)	
High	1363(19.79)	603(25.14)	598(20.49)	73(19.38)	89(13.95)	
Hypertension, *n* (%)	2984(43.33)	972(33.85)	1245(33.44)	325(74.86)	442(75.34)	< 0.0001
Hyperlipidemia, *n* (%)	4954(71.93)	1812(68.01)	2259(67.70)	367(86.12)	516(89.31)	< 0.0001
Heart failure	264(3.83)	52(1.41)	96(2.00)	46(10.06)	70(9.92)	< 0.0001
Ischemic heart disease	498(7.24)	145(4.04)	193(4.58)	64(13.88)	96(15.78)	< 0.0001
Myocardial infarction	361(5.24)	103(3.04)	135(3.17)	47(8.65)	76(12.97)	< 0.0001
TC (mg/dL)	200.23 ± 0.70	202.96 ± 1.41	198.56 ± 0.80	195.99 ± 3.28	198.94 ± 2.53	0.05
TG (mg/dL)	142.74 ± 2.53	131.79 ± 3.96	141.15 ± 3.68	186.56 ± 12.54	181.56 ± 7.43	< 0.0001
LDL-C (mg/dL)	115.77 ± 0.84	117.06 ± 1.45	115.88 ± 1.42	106.44 ± 2.70	115.34 ± 2.87	0.01
HbA1C (%)	5.47 ± 0.02	5.28 ± 0.01	5.27 ± 0.01	6.87 ± 0.10	7.11 ± 0.11	< 0.0001
Fasting glucose (mg/dL)	5.65 ± 0.04	5.28 ± 0.03	5.29 ± 0.02	8.37 ± 0.21	8.11 ± 0.23	< 0.0001
eGFR (mL/min/1.73 m^2^)	93.13 ± 0.66	92.21 ± 0.83	96.55 ± 0.62	80.17 ± 1.29	82.12 ± 1.38	< 0.0001
Waist circumference (cm)	97.53 ± 0.45	95.12 ± 0.60	96.76 ± 0.46	110.20 ± 1.19	110.26 ± 1.27	< 0.0001
Energy intake (Kcal)	2117.02 ± 15.62	2086.27 ± 22.12	2196.58 ± 18.81	1889.66 ± 51.62	1862.77 ± 43.56	< 0.0001
DAPT	8(0.12)	2(0.02)	2(0.04)	2(0.17)	2(0.47)	< 0.0001
Statin therapy	1073(15.59)	322(10.19)	353(9.66)	175(45.09)	223(41.06)	< 0.0001

Data are presented as weighted means ± SEs for continuous variables and unweighted numbers (weighted percentages) for categorical variables.

Low MIND score, the MIND score ≤ 8; High MIND score, the MIND score > 8.

*MIND diet* Mediterranean-DASH Diet Intervention for Neurodegenerative Delay diet, *DM* Diabetes mellitus, *BMI* Body mass index, *TC* Total cholesterol, *TG* Triglyceride, *LDL-C* Low-density lipoprotein cholesterol, *HDL-C* High-density lipoprotein cholesterol, *HbA1c* Glycosylated hemoglobin, type A1C; *eGFR* estimated glomerular filtration rate, *DAPT* Dual antiplatelet therapy.

### The MIND diet and mortality in patients with T2DM

**Fig. 2:**
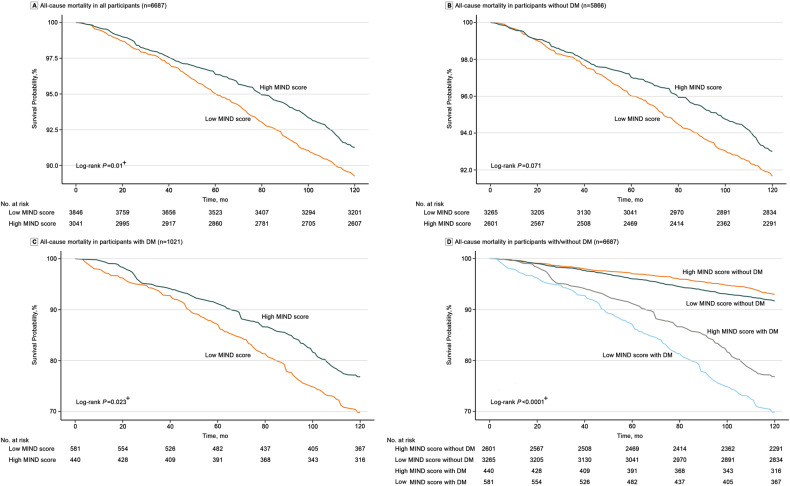
Kaplan-Meier curves for the all-cause mortality in groups of low MIND diet score and high MIND diet score among the whole population (**A**), participants without T2DM (**B**), and patients with T2DM (**C**). Kaplan-Meier curves for the all-cause mortality in groups of MIND diet score-Low/non-DM, MIND diet score -High/non-DM, MIND diet score-Low/DM, and MIND diet score-High/DM were presented in (**D**). ^+^*P* < 0.05. DM Diabetes mellitus, MIND diet Mediterranean-DASH Diet Intervention for Neurodegenerative Delay diet; NHANES, National Health, and Nutrition Examination Surve.

**Fig. 3:**
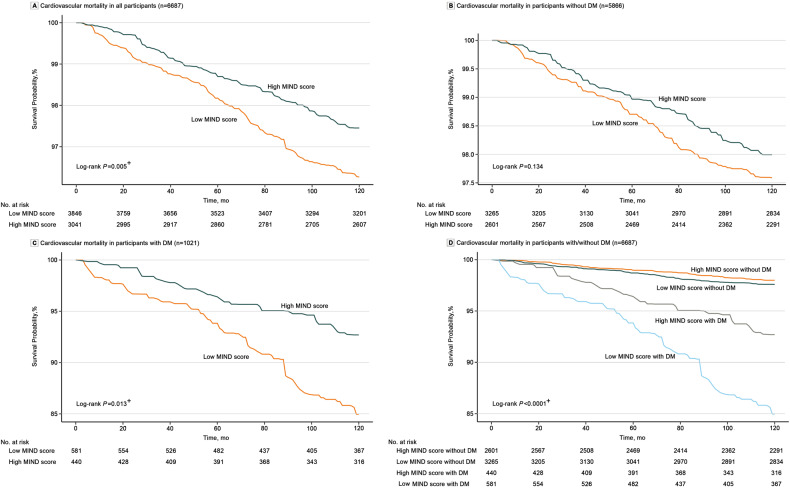
Kaplan-Meier curves for CV mortality in groups of low MIND diet score and high MIND diet score among the whole population (**A**), participants without T2DM (**B**), and patients with T2DM (**C**). Kaplan-Meier curves for CV mortality in groups of MIND diet score-Low/non-DM, MIND diet score -High/non-DM, MIND diet score-Low/DM, and MIND diet score-High/DM were presented in (**D**). ^+^*P* < 0.05. CV Cardiovascular, DM Diabetes mellitus, MIND diet Mediterranean-DASH Diet Intervention for Neurodegenerative Delay diet; NHANES, National Health, and Nutrition Examination Surve.

During a median follow-up of 10 years, 1087 all-cause deaths (338 in T2DM patients) and 377 CV deaths (130 in T2DM patients) were recorded. Subjects with high MIND scores had a significantly reduced risk of all-cause death (Fig. 2C, *P* = 0.023) and CV death (Fig. 3C: *P* = 0.013) in patients with T2DM, but this was not significant in participants without DM (all-cause mortality: *P* = 0.071 [Fig. 2B]; CV mortality: *P* = 0.134 [Fig. 3B]). Figures 1D, 2D show that the presence of both T2DM and a low MIND score predicted the worst prognosis with the highest risk of all-cause (*P* < 0.001) and CV (*P* < 0.001) death.

Cox regression model analysis for the association of the MIND score with the final prognosis was to evaluate participants with and without T2DM. The results in Table 2 showed that T2DM patients with high MIND scores presented a significantly lower risk of all-cause death (HR = 0.75, 95% CI: 0.59, 0.96, *P* = 0.021) and CV death (HR = 0.50, 95% CI: 0.29, 0.87, *P* = 0.014) than those with low MIND score. When focused on the non-T2DM population, a lower risk of all-cause death (HR = 0.83, 95% CI: 0.70, 0.99, *P* < 0.001) was presented in participants with high MIND score, although no significant difference was observed in the risk of CV death between the high MIND score group and low MIND score group (Table 3). In Table 4, we presented the impact of the MIND diet on the life expectancy of the whole cohort. The results showed that participants with high MIND scores presented a significantly lower risk of death compared with those with low MIND scores (Table 4 and Figs. 1A, 2A).

**Table 3:** Cox regression analysis for comparing the risk of all-cause and cardiovascular mortality between groups of low MIND diet score and high MIND diet score in T2DM and non-DM cohorts.

Model	HR (95% CI)				*P*-value^1^	*P*-value^2^
	MIND diet score-L/non-DM	MIND diet score -H/non-DM	MIND diet score -L/DM	MIND diet score -H/DM		
**All-cause mortality**						
Number of deaths/totals	436/3265	313/2601	214/581	124/440		
Crude	Ref [1]	0.83 (0.67, 1.03)	Ref [2]	0.73 (0.56, 0.95)	0.093	0.020
Model 1	Ref [1]	0.63 (0.53, 0.77)	Ref [2]	0.64 (0.50, 0.83)	< 0.001	< 0.001
Model 2	Ref [1]	0.83 (0.67, 0.97)	Ref [2]	0.76 (0.60, 0.96)	0.021	0.022
Model 3	Ref [1]	0.83 (0.70, 0.99)	Ref [2]	0.75 (0.59, 0.96)	< 0.001	0.021
**Cardiovascular mortality**						
Number of deaths/totals	143/3265	104/2601	88/581	42/440		
Crude	Ref [1]	0.83 (0.64, 1.07)	Ref [2]	0.47 (0.26, 0.84)	0.146	0.011
Model 1	Ref [1]	0.62 (0.48, 0.81)	Ref [2]	0.43 (0.24, 0.78)	<0.001	0.005
Model 2	Ref [1]	0.81 (0.60, 1.10)	Ref [2]	0.49 (0.28, 0.86)	0.185	0.012
Model 3	Ref [1]	0.85 (0.62, 1.16)	Ref [2]	0.50 (0.29, 0.87)	0.309	0.014

Model 1: adjusted for age, sex, and race/ethnicity;

Model 2: further adjusted (from Model 1) for education level, family income to poverty ratio, smoking status, BMI, and physical activity;

Model 3: further adjusted (from Model 2) for hypertension, dyslipidemia, energy intake, and eGFR.

Low MIND score, the MIND score ≤ 8; High MIND score, the MIND score > 8.

*HRs* Hazard ratios, *CIs* Confidence intervals, *MIND diet* Mediterranean-DASH Diet Intervention for Neurodegenerative Delay diet, *DM* Diabetes mellitus, *BMI* Body mass index, *eGFR* Estimated glomerular filtration rate, *Ref* Reference, *P* value^1^: The *P* value between MIND diet score-Low/non-DM and MIND diet score-High/non-DM groups. *P* value^2^: The *P* value between MIND diet score-Low/DM and MIND diet score-High/DM groups.

**Table 4:** Cox regression analysis for the risk of all-cause and cardiovascular mortality according to the MIND diet score among all patients.

Model	Per one-score increase in the MIND diet score HR (95% CI)	MIND diet score HR (95% CI)	
		Low	High
**All-cause mortality**			
Number of deaths/totals	1087/6887	650/3846	437/3041
Crude	0.92 (0.87, 0.97)	1.00	0.80 (0.66, 0.97)
Model 1	0.82 (0.78, 0.86)	1.00	0.63 (0.54, 0.73)
Model 2	0.90 (0.86, 0.95)	1.00	0.78 (0.68, 0.91)
Model 3	0.91 (0.86, 0.95)	1.00	0.80 (0.69, 0.92)
**Cardiovascular mortality**			
Number of deaths/totals	377/6887	231/3846	146/3041
Crude	0.88 (0.80, 0.95)	1.00	0.68 (0.50 0.91)
Model 1	0.78 (0.70, 0.86)	1.00	0.53(0.40, 0.71)
Model 2	0.86 (0.78, 0.96)	1.00	0.67 (0.51, 0.89)
Model 3	0.87 (0.79, 0.96)	1.00	0.69 (0.53, 0.91)

Model 1: adjusted for age, sex, and race/ethnicity;

Model 2: further adjusted (from Model 1) for education level, family income to poverty ratio, smoking status, BMI, and physical activity;

Model 3: further adjusted (from Model 2) for diabetes, hypertension, dyslipidemia, energy intake, and eGFR.

Low MIND score, the MIND score ≤ 8; High MIND score, the MIND score > 8.

*HRs* Hazard ratios, *CIs* Confidence intervals, *MIND diet* Mediterranean-DASH Diet Intervention for Neurodegenerative Delay diet, *BMI* Body mass index, *eGFR* estimated glomerular filtration rate.

For comparisons among the four groups, we set the risk of all-cause and CV death in participants with high MIND score/non-DM as the reference. The results in the fully adjusted model showed that compared with participants in the high MIND score/non-DM group, those in low MIND score/non-DM (HR = 1.24, 95% CI: 1.05, 1.48), high MIND score/DM (HR = 1.58, 95% CI: 1.25, 1.98), and low MIND score/DM (HR = 2.00, 95% CI: 1.61, 2.48) groups developed a significantly higher risk of all-cause death (Table S2). KM plots also presented that participants in the low MIND score/DM group showed the highest risk of all-cause and CV death among the four groups (Figs. 2D and 3D). The correlation of the MIND score with FBG, HbA1C, and low-density lipoprotein cholesterol (LDL-C) levels is presented in Table S3, and no significant linear relationship was found.

### Subgroup and sensitivity analysis

Tables S4, S5 present the subgroup and sensitivity analysis. The subgroup analysis showed a significant interaction between the MIND diet and advanced age (age > 65 years, *P* for interaction < 0.001), while no significant interactions were found in other stratifying variables.

After removing non-Hispanic Black participants, people who died within a year of being followed up with, people whose family income to poverty ratio was unknown, people with heart failure, IHD, cerebral disease, dual antiplatelet therapy, statin therapy, and people who were receiving hypoglycemic medication, the results remained consistent.

## Discussion

In this cohort study based on NHANES data, we analyzed 6887 participants (including 1021 patients with T2DM) and conducted a clinical follow-up over a median duration of 10 years. Our study yielded the following key findings: First off, among those with T2DM, higher MIND diet adherence was substantially linked to a lower risk of death from all causes and CVD. Secondly, the protective effect of the MIND diet on prognosis was also observed in the overall population, but not in the non-DM population. These conclusions remained consistent after performing subgroup and sensitivity analyses.

The MIND diet has showed various protective roles, such as improving cognitive performance [15], enhancing physical function including muscle strength [18], and reducing the risk of breast cancer [19]. In relation to its impact on T2DM, a recent study involving 960 participants with T2DM reported that the MIND diet slowed the decline in global cognition and executive function [16]. Furthermore, scholars found a potential association between a higher MIND score and lower blood glucose levels, although the difference was not statistically significant (participants with MIND scores of 7.5–8.0 versus 6.0–7.5 versus < 6.0: 106 ± 27.8 versus 110 ± 61.2 versus 107 ± 31.5, *P*-value = 0.47) [20]. The MIND diet shows promise as a potential therapeutic approach to preventing the development of DM. However, to date, no studies made well-elucidations. The MED diet, which forms a significant component of the MIND diet, has been extensively studied in this context. Controlled trials have demonstrated that the MED diet alleviated traditional CV risk factors, including reductions in blood pressure, triglyceride levels, and glucose levels [21, 22]. Long-term reductions in HbA1c levels have also been observed in individuals with DM following adherence to the MED diet [22, 23]. Similar protective effects were also reported in research focusing on the DASH diet [5]. Given that the MIND diet incorporates the MED and DASH diets components and demonstrates several advantages for individuals with DM, it is reasonable to hypothesize that the MIND diet has similar beneficial effects. This study established an inverse association between the MIND diet and the death risk in T2DM patients, suggesting that the MIND diet represents an anti-diabetic dietary pattern.

In addition to its beneficial effects in T2DM patients, the MIND diet has also demonstrated significant value in lingering life expectancy. A recent cohort study included 882 older participants and found an inverse relationship between the MIND score and death risk. The risk of all-cause death was shown to decrease by 12% per unit increase in MIND score during a 12-year follow-up [24]. Furthermore, the CV benefits of adhering to the MIND diet have been highlighted in previous research. In a rigorous prospective cohort study by Mahdieh et al., involving 2863 participants, the impact of the MIND diet on the risk of CV diseases (including coronary heart disease, stroke, and CV mortality) was investigated. The results indicated that a higher MIND score was associated with a reduced risk of CV disease [25]. Additionally, Asma et al. conducted a case-control study with 193 hospitalized stroke cases and 195 hospital-based controls, revealing an inverse relationship between the MIND score and the risk of stroke [26]. Consistent with these previous studies, the present study also identified a significant inverse association between the MIND score and the death risk in the general population, again emphasizing the MIND diet as a healthy dietary pattern.

Subgroup analysis revealed that the benefits of the MIND diet were significantly amplified among older participants (age > 65 years). This finding aligns with a previous study that specifically examined older individuals (mean age 69.5 years) and reported the improved prognosis roles of the MIND diet in this population [24]. Thus, the MIND diet may hold particular therapeutic value for older adults, and further studies are warranted to validate these observations.

Among the components of the MIND diet, it is likely that whole grains, green leafy vegetables, and beans contribute the most to its protective effects. These components have been shown to enhance glycemic tolerance, improve lipid profiles, and reduce inflammation [25]. Additionally, the MIND diet’s restrictions on fast/fried foods, sweets/pastries, and butter/margarine play a role in its protective benefits for individuals with hyperglycemia. These restrictions limit the consumption of substances known to contribute to metabolic disorders, such as added sugar, saturated fatty acids, and trans fatty acids [27]. Further investigation is needed to elucidate this aspect.

IHD is widely recognized as a potential contributor to poor prognosis, and its connection to glucose metabolism and endothelial function has been extensively studied. Recent observational research has confirmed the role of insulin resistance in the progression of IHD across all stages in individuals with normal glucose tolerance, highlighting the significance of glucose metabolism in IHD development [28]. Furthermore, endothelial function has been shown to impact the prognosis of IHD patients. A rigorous randomized controlled trial demonstrated that reducing coronary endothelial dysfunction through metformin therapy was associated with a decreased CV risk in IHD patients [29]. Overall, significant interactions between IHD and glucose metabolism have been well-documented. To mitigate the impact of IHD on prognosis, we performed a sensitivity analysis excluding participants with IHD including MI. The results consistently aligned with the main conclusions, further reinforcing the association between adherence to the MIND diet and reduced risk of mortality. It is important to note that the NHANES dataset used in this study did not provide detailed information on the specific type of MI. Therefore, the current analysis was unable to examine the association of the MIND diet with the prognosis in patients with different types of MI. Future studies are encouraged to investigate this relationship and explore the potential impact of the MIND diet on the prognosis of patients with various types of MI.

Diabetes can lead to inflammation and oxidative stress, which can adversely affect coronary plaques, leading to CV events and death [30]. Hypoglycemic treatments have been reported to reduce CV risk in diabetic patients. In a multi-center study, scholars reported that hypoglycemic therapy like sodium/glucose cotransporter 2 inhibitors (SGLT2-I) was significantly associated with the reduced risk of major adverse cardiac events [31]. Besides, a recent rigorous study also showed that SGLT2i treatment in T2DM is associated with a reduced incidence of in-stent restenosis (ISR) in patients undergoing percutaneous coronary intervention [32]. To minimize the potential impact of hypoglycemic treatment on the prognosis of individuals with diabetes, we conducted a sensitivity analysis excluding participants receiving such therapy. The results demonstrated a significant and robust association between adherence to the MIND diet and a reduced risk of CV mortality. It is important to note that the specific hypoglycemic drugs used and the atherosclerotic plaque stability were not reported due to data limitations in the NHANES dataset. Considering the widely reported CV benefits of SGLT2-I therapy, it would be valuable to investigate the association between the MIND diet and CV mortality by taking into account the potential involvement of SGLT2-I therapies in future studies. Besides, the effect of the MIND diet on atherosclerotic plaque stability was also another interest to be focused on in the future study.

In addition to the hypoglycemic treatment, DAPT and statin therapy were also associated with improved prognosis in diabetic patients [33, 34]. These therapies exert their effects through mechanisms such as anti-thrombotic effects and reduced inflammation in atherosclerotic plaques, leading to plaque stabilization characterized by thickened fibrous caps and macrocalcification [35, 36]. To minimize the potential influence of statins or DAPT treatment on long-term mortality risk, we performed a sensitivity analysis excluding subjects receiving these treatments. The results remained consistent, further reinforcing the robustness of our findings.

Firstly, the diagnosis of T2DM and assessment of MIND scores were based on self-reported questionnaires without verification by specialists, which introduces the possibility of bias. Secondly, the calculation of the MIND score relied solely on food data from an FFQ, and the 24 h recall food data from NHANES could not be used due to differences in units of measurement. This limitation may have affected the accuracy of the MIND scores. Thirdly, the association between the MIND diet score and T2DM was examined using cross-sectional analysis, which does not establish a robust causal relationship. Further studies using longitudinal designs are necessary to address this limitation. Fourthly, this study was limited by the unavailability of data on cardiac function and echocardiogram indices, as these measures were not included in the NHANES dataset. The absence of these variables may have introduced bias in the analysis of CV death risk. At last, the lack of randomized performance limits the strength of the conclusions. Therefore, future exploration of the MIND diet in T2DM should be performed in randomized clinical trials.

Generally, the findings of this study provide valuable insights into the potential protective effects of the MIND diet on the prognosis of T2DM patients. Further research is warranted to confirm and expand upon these findings, reinforcing the importance of exploring the role of the MIND diet in improving outcomes for individuals with T2DM.

### Supplementary information

supplementary material

## Data Availability

All data are available at the NHANES website https://www.cdc.gov/nchs/nhanes/index.htm.
