# Supplementary material for: Association Between MIND Diet Adherence and Mortality: Insights from Diabetic and Non-Diabetic Cohorts
Source: Nutr Diabetes. 2023 Oct 10;13:18. doi: 10.1038/s41387-023-00247-1 (PMC10564876; doi:10.1038/s41387-023-00247-1)
Supplement: Supplementary file 1 — supplementary material [file 41387_2023_247_MOESM1_ESM.docx]

**SUPPLEMENTAL MATERIAL**

Text S1. Introduction to the food frequency questionnaire 2

Text S2. Information on study covariates 2

Table S1. The calculation for the MIND scores. 4

Table S2. Cox regression analysis for comparing the risk of all-cause and cardiovascular mortality among groups of MIND diet score -high/non-DM, MIND diet score-low/non-DM, MIND diet score-high/DM, and MIND diet score-low/DM 6

Table S3. Correlation analysis between the MIND diet score and HbA1C, fasting glucose, and LDL-C in patients with T2DM, without T2DM, and whole. 8

Table S4. Subgroup analyses of the associations (HRs, 95% CIs) between the MIND diet score and all-cause mortality among patients according to their MIND diet score and DM status 9

Table S5. Sensitivity Analyses of the associations (HRs, 95% CIs) between the MIND diet score and mortality among patients according to their MIND diet score and DM status 11

References 14

.

.

.

**Text S1.** **Introduction to the food frequency questionnaire**

The food frequency questionnaire (FFQ) was used to collect information on the frequency of food consumption during the past 12 months, which provides information on the frequency covariates on food intake. The calculation of the MIND scores was based on the data of the frequency of food intake in the FFQ such as “How often did you drink wine or wine coolers?”, and the options of this question including never, 1 time per month or less, 2-3 times per month, 1-2 times per week, etc. The National Health and Nutrition Examination Survey (NHANES) FFQ questionnaire was developed by the National Institutes of Health, National Cancer Institute (NCI), which contains several types of questions. There are 151 frequency questions (this includes two frequency sub-questions querying intake over two seasons for questions such as “How often drink tomato juice?” etc.). The FFQ also asks about the proportion (reported as fractions) of the time certain types of foods were eaten over the past 12 months such as sugar-free soft drinks, whole grain foods, and light, low-fat or fat-free varieties of foods. In addition, quality control programs and manual verification checks were used to verify the completeness and accuracy of data.

**Text S2. Information on study covariates**

Information on age, sex, race/ethnicity, education, family income, smoking status, alcohol consumption, physical activity, and self-reported medical conditions were obtained through standardized questionnaires during in-home interviews by trained interviewers. Heights, weights, waist circumferences, blood pressures, and blood samples were collected from physical examinations at MEC using standard protocols.

Race/ethnicity was categorized as non-Hispanic White, non-Hispanic Black, Mexican American, and others. Smoking status was categorized as never (smoked less than 100 cigarettes in life), former (smoked more than 100 cigarettes in life but quit smoke now), and current (smoked more than 100 cigarettes in life and still smoke some days or every day). Education level was categorized as under high school, high school or equivalent, and college or higher. The family income to poverty ratio was classified as ≤1.0, 1.0–3.0, and >3.0. Physical activity was measured as the weekly minutes of moderate and vigorous activities multiplied by the metabolic equivalent (MET) level and divided into four categories: sedentary (without regular physical activity, MET-minutes/week =0), insufficient (0 < MET-minutes/week <500), moderate (500 ≤ MET-minutes/week ≤1000), and high (>1000 MET-minutes/week) (1). Body mass index (BMI, kg/m2) was calculated as weight in kilograms divided by height in meters squared. Hypertension was defined as a self-reported medical history of high blood pressure, receiving antihypertensive drugs, or blood pressure measurement ≥140/90 mmHg (2). Diabetes mellitus was defined as a self-reported medical history of diabetes, receiving oral hypoglycemic agents or insulin, fasting glucose level ≥126 mg/dL, or hemoglobin A1c (HbA1c) level ≥6.5% (3). Hyperlipidemia was defined as serum triglycerides (TG) ≥150 mg/L, total cholesterol (TC) ≥200 mg/dL, low-density lipoprotein cholesterol (LDL-C) ≥130 mg/dL, high-density lipoprotein cholesterol (HDL-C) ≤40 mg/dL in men or ≤50 mg/dL in women, or receiving medication for hyperlipidemia.

The biochemical parameters, including TG, TC, HDL-C, LDL-C, and HbA1c were measured among partial participants who provided blood samples at MEC. The estimated glomerular filtration rate (eGFR, mL/min/1.73m^2^) was computed using the Chronic Kidney Disease Epidemiology Collaboration creatinine equation (3).

**Table S1.** **The calculation for the MIND scores**

| Scores | 0 | 0.5 | 1 |
| --- | --- | --- | --- |
| Green leafy vegetables | ≤2 servings/wk | > 2 to <6 servings/wk | ≥6 servings/wk |
| Other vegetables | <5 servings/wk | 5 – <7 servings/wk | ≥1 serving/day |
| Berries | <1 serving/wk | 1 serving/wk | ≥2 servings/wk |
| Nuts | <1 serving/mo | 1 serving/mo – <5 servings/wk | ≥5 servings/wk |
| Olive Oil | Not primary oil |  | Not primary oil |
| Butter, Margarine | >2 T/d | 1–2 T/d | <1 T/d |
| Cheese | 7+ servings/wk | 1–6 servings/wk | < 1 serving/wk |
| Whole Grains | <1 serving/d | 1–2 servings/d | ≥3 servings/d |
| Fish (not fried) | Rarely | 1–3 meals/mo | ≥1 meal/wk |
| Beans | <1 meal/wk | 1–3 meals/wk | >3 meals/wk |
| Poultry (not fried) | <1 meal/wk | 1 meal/wk | ≥2 meals/wk |
| Red meat and products | 7+ meals/wk | 4–6 meals/wk | < 4 meals/wk |
| Fast fried foods | 4+ times/wk | 1–3 times/wk | <1 time/wk |
| Pastries & Sweets | 7+ servings/wk | 5 −6 servings/wk | <5 servings/wk |
| Wine | >1 glass/d or never | 1 glass/mo – 6 glasses/wk | 1 glass/d |
| Total scores |  |  | 15 |

Green leafy vegetables: kale, collards, greens; spinach; lettuce/tossed salad.

Other vegetables: green/red peppers, squash, cooked carrots, raw carrots, broccoli, celery, potatoes, peas or lima beans, potatoes, tomatoes, tomato sauce, string beans, beets, corn, zucchini/summer squash/eggplant, coleslaw, potato salad.

Berries: strawberries.

Fish (not fried): tuna sandwich, fresh fish as main dish; not fried fish cakes, sticks, or sandwiches

Beans: beans, lentils, soybeans

Poultry (not fried): chicken or turkey sandwich, chicken or turkey as main dish and never eat fried at home or away from home

Red meat and products: cheeseburger, hamburger, beef tacos/burritos, hot dogs/sausages, roast beef or ham sandwich, salami, bologna, or other deli meat sandwich, beef (steak, roast) or lamb as main dish, pork or ham as main dish, meatballs or meatloaf

Fast fried foods: How often do you eat fried food away from home (like French fries, chicken nuggets)

Pastries & Sweets: biscuit/roll, poptarts, cake, snack cakes/twinkies, Danish/sweet rolls/pastry, donuts, cookies, brownies, pie, candy bars, other candy, ice cream, pudding, milkshakes/frappes

**Table S2. Cox regression analysis for comparing the risk of all-cause and cardiovascular mortality among groups of MIND diet score -high/non-DM, MIND diet score-low/non-DM, MIND diet score-high/DM, and MIND diet score-low/DM.**

| **Model** | **HR (95% CI)** | | | | ***P* trend** |
| --- | --- | --- | --- | --- | --- |
|  | **MIND diet score-high/non-DM** | **MIND diet score-low/non-DM** | **MIND diet score-high /DM** | **MIND diet score-low /DM** |  |
| **All-cause mortality** |  |  |  |  |  |
| Number of deaths/totals | 313/2601 | 436/3265 | 124/440 | 214/581 |  |
| Crude | 1.00 | 1.20 (0.97, 1.50) | 3.60 (2.88, 4.52) | 4.94 (3.71, 6.56) | <0.001 |
| Model 1 | 1.00 | 1.58 (1.34, 1.87) | 1.77 (1.40, 2.24) | 2.73 (2.10, 3.55) | <0.001 |
| Model 2 | 1.00 | 1.27 (1.06, 1.52) | 1.66 (1.28, 2.15) | 2.12 (1.67, 2.69) | <0.001 |
| Model 3 | 1.00 | 1.24 (1.05, 1.48) | 1.58 (1.25, 1.98) | 2.00 (1.61, 2.48) | <0.001 |
| **Cardiovascular mortality** |  |  |  |  |  |
| Number of deaths/totals | 104/2601 | 143/3265 | 42/440 | 88/581 |  |
| Crude | 1.00 | 1.21 (0.94, 1.56) | 3.70 (2.44, 5.62) | 7.92 (4.84 12.95) | <0.001 |
| Model 1 | 1.00 | 1.61 (1.25, 2.07) | 1.81 (1.17, 2,79) | 4.16 (2.57, 6.75) | <0.001 |
| Model 2 | 1.00 | 1.24 (0.95, 1.62) | 1.54 (0.90, 2.63) | 3.18 (2.02, 5.01) | <0.001 |
| Model 3 | 1.00 | 1.19 (0.90, 1.57) | 1.39 (0.82, 2.35) | 2.82 (1.81, 4.40) | 0.002 |

Model 1: adjusted for age, sex, and race/ethnicity;

Model 2: further adjusted (from Model 1) for education level, family income to poverty ratio, smoking status, BMI, and physical activity;

Model 3: further adjusted (from Model 2) for hypertension, dyslipidemia, energy intake, and eGFR.

Low MIND score, the MIND score ≤8; High MIND score, the MIND score >8.

Abbreviations: HRs, hazard ratios; CIs, confidence intervals; MIND diet, Mediterranean-DASH Diet Intervention for Neurodegenerative Delay diet; DM, diabetes mellitus; BMI, body mass index; eGFR, estimated glomerular filtration rate.

**Table S3. Correlation analysis between the MIND diet score and HbA1C, fasting glucose and LDL-C in patients with T2DM, without T2DM and whole**

| **Variables** | **β** | ***P* value** |
| --- | --- | --- |
| **Whole cohort** |  |  |
| Fasting glucose (mg/dL)  HbA1c, % | -0.005 | 0.816 |
|  | -0.004 | 0.675 |
| LDL-C, mmol/L | 0.258 | 0.753 |
| **Non-T2DM patients** |  |  |
| Fasting glucose (mg/dL)  HbA1c, % | -0.001 | 0.869 |
|  | 0.005 | 0.321 |
| LDL-C, mmol/L | 0.721 | 0.414 |
| **T2DM patients** |  |  |
| Fasting glucose (mg/dL)  HbA1c, % | -0.075 | 0.061 |
|  | -0.011 | 0.917 |
| LDL-C, mmol/L | -2.871 | 0.044 |

Abbreviations: MIND diet, Mediterranean-DASH Diet Intervention for Neurodegenerative Delay diet; DM, diabetes mellitus; HbA1c, glycosylated [hemoglobin A1c;](http://www.dictall.com/indu/214/213618925C9.htm) LDL-C, low-density lipoprotein cholesterol

**Table S4. Subgroup analyses of the associations (HRs, 95% CIs) between the MIND diet score and all-cause mortality among patients** **according to their MIND diet score and DM status**

| **Subgroup** | **No. deaths/Total** | **MIND diet score-high/non-DM** | **MIND diet score-low/non-DM** | **MIND diet score-high /DM** | **MIND diet score-low /DM** | ***P* for interaction** |
| --- | --- | --- | --- | --- | --- | --- |
| Age (years) |  |  |  |  |  | <0.001 |
| <60 | 145/4284 | 1.00 | 1.08 (0.69, 1.70) | 5.41 (3.30, 9.64) | 2.85 (1.34, 6.08) |  |
| ≥60 | 942/2603 | 1.00 | 1.25 (0.98, 1.59) | 1.12 (0.80, 1.58) | 1.89 (1.48, 2.40) |  |
| Sex |  |  |  |  |  | 0.509 |
| Male | 627/3181 | 1.00 | 1.10 (0.86, 1.41) | 1.39 (0.93, 2.07) | 1.78 (1.31, 2.42) |  |
| Female | 460/3706 | 1.00 | 1.44 (1.12, 1.85) | 1.90 (1.26, 2.88) | 2.25 (1.57, 3.21) |  |
| Race/ethnicity |  |  |  |  |  | 0.676 |
| White | 742/3809 | 1.00 | 1.23 (1.01, 1.49) | 1.67 (1.22, 2.27) | 1.92 (1.50, 2.47) |  |
| Non-White | 345/3078 | 1.00 | 1.39 (0.89, 2.18) | 1.34 (0.72, 2.49) | 2.45 (1.39, 4.33) |  |
| Smoking status |  |  |  |  |  | 0.165 |
| Never | 406/3526 | 1.00 | 1.51 (1.12, 2.05) | 2.18 (1.36, 3.50) | 2.33 (1.55, 3.51) |  |
| Former/Current | 681/3361 | 1.00 | 1.19 (0.98, 1.44) | 1.31 (0.89, 1.93) | 1.91 (1.51, 2.42) |  |
| Hypertension |  |  |  |  |  | 0.514 |
| Yes | 787/2984 | 1.00 | 1.24 (1.07, 1.44) | 1.48 (1.17, 1.88) | 2.00 (1.63, 2.44) |  |
| No | 300/3903 | 1.00 | 1.35 (0.93, 1.95) | 2.15 (0.93, 4.93) | 1.87 (1.11, 3.16) |  |
| BMI, kg/m^2^ |  |  |  |  |  | 0.573 |
| <30 | 730/4438 | 1.00 | 1.31 (1.08, 1.59) | 1.43 (1.01, 2.03) | 1.79 (1.32, 2.42) |  |
| ≥30 | 316/2341 | 1.00 | 1.13 (0.76, 1.68) | 1.59 (0.90, 2.83) | 2.19 (1.37, 3.47) |  |
| Physical activity |  |  |  |  |  | 0.389 |
| Sedentary/Insufficient | 807/4395 | 1.00 | 1.29 (1.06, 1.56) | 1.45 (1.12, 1.88) | 1.98 (1.53, 2.55) |  |
| Moderate/High | 280/2492 | 1.00 | 1.11 (0.74, 1.66) | 2.01 (1.07, 3.78) | 2.35 (1.69, 3.27) |  |
| eGFR, mL/min/1.73m^2^ |  |  |  |  |  | 0.144 |
| ≤90 | 856/3024 | 1.00 | 1.34 (1.08, 1.66) | 1.49 (1.11, 2.00) | 1.92 (1.46, 2.53) |  |
| >90 | 231/3863 | 1.00 | 0.99 (0.58, 1.69) | 2.08 (1.17, 3.70) | 2.83 (1.52, 5.27) |  |

All the models adjusted for age, sex, race/ethnicity, education level, family income to poverty ratio, smoking status, BMI, physical activity, hypertension, dyslipidemia, energy intake, and eGFR, with exception of stratifying factors.

Low MIND score, the MIND score ≤8; High MIND score, the MIND score >8.

Abbreviations: HRs, hazard ratios; CIs, confidence intervals; MIND diet, Mediterranean-DASH Diet Intervention for Neurodegenerative Delay diet; DM, diabetes mellitus; BMI, body mass index; eGFR, estimated glomerular filtration rate.

**Table S5. Sensitivity Analyses of the associations (HRs, 95% CIs) between the MIND diet score and CV mortality among patients according to their MIND diet score and DM status**

| **Analysis** | **MIND diet score-high/non-DM** | **MIND diet score-low/non-DM** | **MIND diet score-high /DM** | **MIND diet score-low /DM** | ***P* trend** |
| --- | --- | --- | --- | --- | --- |
| **Excluding non-Hispanic Black participants (N=5474)** |  |  |  |  |  |
| All-cause mortality | 1.00 | 1.21 (1.00, 1.46) | 1.65 (1.25, 2.19) | 2.03 (1.58, 2.60) | <0.001 |
| Cardiovascular mortality | 1.00 | 1.24 (0.92, 1.68) | 1.44 (0.78, 2.67) | 2.85 (1.78, 4.58) | <0.001 |
| **Excluding participants who died within one year of follow-up (N=6712)** |  |  |  |  |  |
| All-cause mortality | 1.00 | 1.29 (1.09, 1.53) | 1.67 (1.32, 2.21) | 2.01 (1.65, 2.46) | <0.001 |
| Cardiovascular mortality | 1.00 | 1.18 (0.90, 1.53) | 1.38 (0.81, 2.37) | 2.61 (1.67, 4.09) | <0.001 |
| **Excluding participants with missing data on family income to poverty ratio (N=6471)** |  |  |  |  |  |
| All-cause mortality | 1.00 | 1.24 (1.04, 1.47) | 1.60 (1.28, 2.00) | 2.00 (1.62, 2.47) | <0.001 |
| Cardiovascular mortality | 1.00 | 1.24 (0.93, 1.65) | 1.37 (0.80, 2.35) | 2.70 (1.76, 4.15) | <0.001 |
| **Excluding** **participants with** **heart failure (N=6623)** |  |  |  |  |  |
| All-cause mortality | 1.00 | 1.21(1.00, 1.48) | 1.47(1.08, 1.99) | 1.63(1.22, 2.17) | <0.001 |
| Cardiovascular mortality | 1.00 | 1.12(0.75, 1.67) | 1.51(0.79, 2.91) | 2.33(1.32, 4.14) | 0.004 |
| **Excluding participants with ischemic heart disease (N=6389)** |  |  |  |  |  |
| All-cause mortality | 1.00 | 1.17(0.96, 1.43) | 1.46(1.11, 1.94) | 1.82(1.46, 2.27) | 0.011 |
| Cardiovascular mortality | 1.00 | 1.04(0.79, 1.37) | 1.32(0.77, 2.28) | 2.61(1.57, 4.36) | 0.011 |
| **Excluding** **participants with DAPT (N=6879)** |  |  |  |  |  |
| All-cause mortality | 1.00 | 1.25(1.04, 1.52) | 1.54(1.15, 2.07) | 2.00(1.52, 2.62) | <0.001 |
| Cardiovascular mortality | 1.00 | 1.21(0.85, 1.73) | 1.45(0.80, 2.63) | 3.00(1.85, 4.84) | <0.001 |
| **Excluding** **participants with statin therapy (N=5814)** |  |  |  |  |  |
| All-cause mortality | 1.00 | 1.19(0.96, 1.47) | 1.44(0.96, 2.18) | 1.96(1.43, 2.69) | <0.001 |
| Cardiovascular mortality | 1.00 | 0.87(0.58, 1.30) | 1.12(0.52, 2.38) | 2.34(1.33, 4.11) | 0.01 |
| **Excluding** **participants with hypoglycemic treatment (N=6257)** |  |  |  |  |  |
| All-cause mortality | 1.00 | 1.24(1.02, 1.50) | 1.86(1.06, 3.26) | 1.75(1.21, 2.55) | <0.001 |
| Cardiovascular mortality | 1.00 | 1.20(0.83, 1.74) | 1.69(0.77, 3.73) | 1.91(0.99, 3.69) | 0.029 |
| **Excluding participants with cerebral diseases (N=6551)** |  |  |  |  |  |
| All-cause mortality | 1.00 | 1.20 (1.01, 1.42) | 1.48 (1.07, 2.06) | 1.96 (1.51, 2.55) | <0.001 |
| Cardiovascular mortality | 1.00 | 1.10 (0.81, 1.49) | 1.26 (0.75, 2.12) | 2.78 (1.68, 4.61) | <0.001 |
| **Excluding participants with missing data on smoking status, BMI, eGFR, and energy intake (N=6152)** |  |  |  |  |  |
| All-cause mortality | 1.00 | 1.25 (1.03, 1.52) | 1.56 (1.17, 2.07) | 1.96 (1.50, 2.56) | <0.001 |
| Cardiovascular mortality | 1.00 | 1.24 (0.86, 1.78) | 1.50 (0.84, 2.67) | 2.94 (1.83,4.71) | <0.001 |

All the models were adjusted for age, sex, race/ethnicity, education level, family income to poverty ratio, smoking status, BMI, physical activity, hypertension, dyslipidemia, energy intake, and eGFR.

Abbreviations: HRs, hazard ratios; CIs, confidence intervals; MIND diet, Mediterranean-DASH Diet Intervention for Neurodegenerative Delay diet; DM, diabetes mellitus; BMI, body mass index; eGFR, estimated glomerular filtration rate; DAPT, dual antiplatelet therapy.

**REFERENCE**

1. Tucker LA. Physical activity and telomere length in U.S. men and women: An NHANES investigation. Preventive medicine 2017;100:145-151

2. Williams B, Mancia G, Spiering W, Agabiti Rosei E, Azizi M, Burnier M, et al. 2018 Practice Guidelines for the management of arterial hypertension of the European Society of Hypertension and the European Society of Cardiology: ESH/ESC Task Force for the Management of Arterial Hypertension. Journal of hypertension 2018;36:2284-2309

3. Skversky AL, Kumar J, Abramowitz MK, Kaskel FJ, Melamed ML. Association of glucocorticoid use and low 25-hydroxyvitamin D levels: results from the National Health and Nutrition Examination Survey (NHANES): 2001-2006. The Journal of clinical endocrinology and metabolism 2011;96:3838-3845
